# Does Political Participation Strengthen the Relationship between Civic Morality and Environmentally Friendly Attitudes? Evidence from South Korea

**DOI:** 10.3390/ijerph19042095

**Published:** 2022-02-13

**Authors:** Jaeyoung Lim, Kuk-Kyoung Moon

**Affiliations:** 1 Department of Public Administration and Social Welfare, Chosun University, Gwangju 61452, Korea; jaeyounglim@yahoo.com; 2 Department of Public Administration, Inha University, Incheon 22212, Korea

**Keywords:** civic morality, political participation, green pricing, green taxation

## Abstract

Environmental degradation in recent years has been threatening not only environmental sustainability but also human viability. To counter these threats, this study focuses on whether civic morality is associated with taxation for environmental protection (green taxation) and with higher pricing for environmental protection (green pricing) and whether the relationship between civic morality and green taxation, as well as that between civic morality and green pricing, is moderated by individuals’ perceptions of their own political participation. Employing the 2014 Korean General Social Survey and an ordered probit model, the study finds that civic morality is positively associated with green pricing and green taxation. Moreover, the positive relationship between civic morality and green pricing, as well as between civic morality and green taxation, is further strengthened when individuals’ perceptions of their own political participation are strong.

## 1. Introduction

Rapidly developing climate change is turning into an all-out environmental crisis that is upending the lives and livelihoods of people worldwide [1]. Humankind has witnessed an unprecedented number of disasters, from uncontrollable wildfires to winter storms, in recent years [2,3]. The Intergovernmental Panel on Climate Change (IPCC) reported that there would be a 1.5 °C increase in temperature between 2030 and 2052, foretelling an ominous future for all countries worldwide; some countries would be uninhabitable, with people fleeing from them for a better climate due to sweltering weather and scarcity of water resources [4]. Given this context, mitigating and adapting to the impact of climate change is more urgent than ever before. In particular, such efforts require significant financial sacrifices from citizens. Thus, this study examines factors that may cause individuals to be willing to support increased taxation (green taxation) and pricing (green pricing) for environmental protection. While green taxation and green pricing may not solve the environmental crisis, they at least provide the government with financial resources to cope with it. Among the possible explanatory factors, this study explores civic morality and political participation and the interplay between the two in explaining green taxation and green pricing.

Civic morality, or citizenship norms, refers to individuals’ voluntary willingness to abide by social rules and norms [5,6]. The obligation to follow social rules and norms is premised upon trust in others and doing good for the public [6]. Thus, individuals with a high degree of civic morality are likely to support communal causes, such as environmental protection. As the current environmental crisis deepens, its mitigation will require significant input and support from citizens. How much citizens would be willing to accept some sacrifices—such as higher taxation and pricing—for such causes is a crucial component of policymakers’ calculations to ensure that the world remains environmentally sustainable and viable for human life.

The study also investigates the impact of political participation on green taxation and green pricing. Touching upon perceptions of participation in politics, political participation increasingly takes on an important dimension for furthering environmental causes. We posit that political participation nurtures individuals’ sense of self-control and self-competence, and that these lead individuals to be actively involved in bringing about desirable social or political change [7,8,9]. Mitigating environmental problems can be considered a desirable outcome, which individuals with a high degree of political participation may be interested in fulfilling. Thus, individuals’ sense of political participation is expected to be positively associated with their support for green taxation and green pricing.

More importantly, the study examines whether the relationship between individuals’ sense of civic morality and their support for green taxation and pricing is moderated by their perceptions of political participation. We argue that because both civic morality and political participation serve as functional equivalents due to their positive impact on individuals’ support for green taxation and green pricing, political participation can strengthen the positive link between civic morality and green taxation and between civic morality and green pricing.

Focusing on the joint effects of the perceptual factors—civic morality and political participation—this study aims to enrich our understanding of green taxation and green pricing. Given that these efforts can be categorized as environmentally friendly attitudes, the study can shed light on the factors that help mitigate the impact of adverse human actions on climate change facing the world today. Moreover, it is crucial to understand the mechanisms that link human values to tangible actions. By emphasizing the moderation effect of political participation, the study helps uncover the value–action gap that addresses why some values do not lead to actions [10,11]. Using political participation as a factor in linking values (civic morality) to green taxation and green pricing, this study adds to the understanding of the linkages between human values and actions. 

This article proceeds as follows. First, we will explore the concepts of civic morality and political participation and how they can be associated with green taxation and green pricing. Then, we will examine the moderation of political participation on the relationship between civic morality and green taxation, and between civic morality and green pricing and the mechanism behind these relationships. Then, we will empirically test the hypotheses generated by earlier theoretical explorations. Finally, we discuss the results and their implications, along with their limitations.

## 2. Civic Morality and Environmentally Friendly Attitudes

Civic morality plays a vital role in sustaining a democratic policy [6]. Also known as citizenship norms, civic morality is defined as having a sense of duty through which individuals voluntarily comply with social rules and norms, even if these may be unpopular [5]. Civic morality is critical to upholding social order and tranquility in an increasingly polarized world [6]. Individuals with a high degree of civic morality are willing to adhere to social rules and norms because doing so, they believe, strengthens public interest [6]. As such, civic morality helps amplify public gains rather than private gains. Individuals with a high degree of civic morality feel compelled to maintain integrity and respect toward other members of society and to help reduce law enforcement and deterrence expenses incurred by unlawful actions of citizens [5,6]. 

Scholars have noted that being a good citizen requires following a wide array of norms, such as voting regularly, legal compliance, active involvement in community affairs, and support of the socially disadvantaged [6,12]. Some rules, of course, are unpopular, yet individuals with a high degree of civic morality follow them, believing that their actions help facilitate the public good [6]. Thus, civic morality can explain why individuals keep paying their tax bills [13]. It is true that such legal compliance may have stemmed from economic calculations, but individuals imbued with civic considerations take the moral and ethical dimensions of their lives as citizens seriously. 

Several studies have noted positive connections between individuals with a high degree of civic morality and their attitudes toward pro-social behaviors [13,14,15]. One study points to a positive linkage between individuals with a high degree of tax morale—synonymous with civic morality and applied to an area of taxation—and tax compliance [13]. As civic morality is closely associated with the public good, it is not surprising to find positive connections between civic morality and environmental causes. For instance, individuals who are exposed to environmental civic duty during their school years are more likely to be supportive of sustainable development [14], and individuals with a high degree of environmental citizenship tend to exhibit environmentally friendly behaviors [15]. Individuals who care about civic cooperation are likely to display positive attitudes toward taxation for environmental protection [16]. 

These studies point to the likelihood of a positive link between civic morality and green pricing, as well as between civic morality and green taxation, as green pricing and green taxation are increasingly being considered a public good in the climate crisis facing the world. Thus, the following hypotheses are advanced for empirical investigation.

**Hypothesis** **1.** *Individuals’ evaluations of their own civic morality are positively associated with their support for green pricing*.

**Hypothesis** **2.** *Individuals’ evaluations of their own civic morality are positively associated with their support for green taxation*.

## 3. Political Participation as the Moderator of the Link between Civic Morality and Environmentally Friendly Attitudes

Political participation is an essential part of democratic governance. Defined as voluntary individual participation in influencing politics, political participation is increasingly forming a vital component of contemporary political life, in which citizens are asked to partake actively in important political and administrative matters [8]. Political participation also stimulates a virtuous cycle in which active political participation helps sustain and strengthen democratization [17]. 

Political participation engenders self-actualization, with which citizens reshape personal and political outcomes [7,9,17]. Self-actualization is possible because of political efficacy [18]. Political efficacy itself can be defined as a sense of being able to influence political change and is known to help individuals attain self-control and self-competence [18]. Thus, political participation helps individuals nurture their political efficacy, which, in turn, motivates them to challenge and resolve a wide array of social and political issues [19,20]. Scholars have noted that political efficacy consists of two components: internal and external efficacy [19,21,22]. Internal efficacy is concerned with individuals’ sense of self-competence in understanding important political issues of the day and influencing political change, whereas external efficacy deals with individuals’ perceptions of the degree to which the government responds to their demands [19,21,22]. These two concepts are not separate from each other; rather, they reinforce each other [23,24]. According to Bandura, internal efficacy enables individuals to pursue collective efficacy [23,24]. When people possess a significant degree of self-control and self-competence, they are likely to share what they believe with others and work collectively to solve political and social problems [23,24]. In other words, individuals experience a high degree of personal agency, which enables them to pursue collective actions. Thus, political participation offers a mechanism through which individuals can experience a sense of self-efficacy, which motivates them to pursue the resolution of communal problems, such as what to do for environmental protection and mitigation of the climate crisis.

Several studies have confirmed the positive link between political participation and communal efforts, such as supporting actions to address environmental problems [25,26,27]. Some monumental laws to protect the environment, such as the Clean Air Act (1970) in the United States during the 1970s, were byproducts of political rallies that reflected the active political participation of citizens [28]. Environmental efforts in South Korea have also reflected citizens’ political activism to address the harmful effects of toxicants, such as phenol contamination in drinking water, during the 1990s [29,30]. Indeed, citizens’ political activism has made environmental reforms possible over the last several decades.

More importantly, this study focuses on the moderation of political participation in the link between individuals’ perceptions of civic morality and their support for green taxation and green pricing. Political participation enables citizens to experience a sense of self-competence that contributes to influencing communal outcomes, such as environmental protection, for which green taxation and green pricing are increasingly necessary. Individuals’ perceptions of civic morality are also positively associated with their views toward green taxation and green pricing because civic morality makes individuals willing to comply with desirable social norms and obligations, of which environmental protection is an urgent contemporary agenda. Political participation and civic morality, then, serve to positively reinforce each other in their pursuit of positive political or social outcomes. In other words, they can work as functional equivalents. Thus, it is highly likely that individuals with a greater level of civic morality are more likely to show appreciation and support for green taxation and green pricing when they perceive themselves as having a greater level of political participation. Thus, the following hypotheses are presented for empirical testing:

**Hypothesis** **3.** *Political participation moderates the relationship between individuals’ perceptions of civic morality and their support for green pricing, such that the relationship becomes stronger as the level of participation increases*.

**Hypothesis** **4.** *Political participation moderates the relationship between individuals’ perceptions of civic morality and their support for green taxation, such that the relationship becomes stronger as the level of participation increases*.

**Figure 1:**
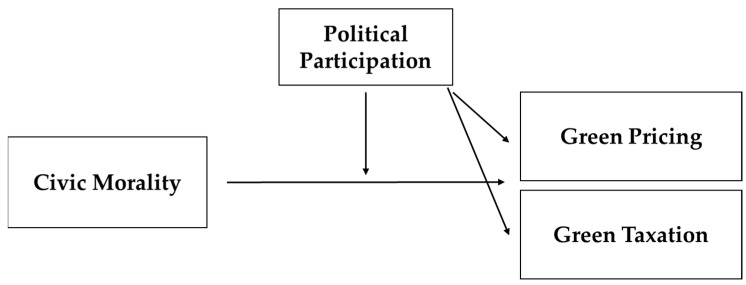
The conceptual framework.

Figure 1 provides a conceptual overview of the empirical model investigated in this study.

## 4. Data Management

The data for this study were derived from the 2014 Korean General Social Survey (KGSS). Beginning in 2003 and carried out by the Sungkyunkwan University Survey Research Center, the survey was the Korean version of the U.S. General Social Survey and followed the latter’s format of asking respondents a select number of the same questions, with every survey supplemented with topical questions repeated in intermittent years [31]. Given the different individuals interviewed for the survey, the KGSS is cross-sectional in nature. The KGSS was available every year between 2003 and 2014 and became available biennially after 2014. We relied on the 2014 survey dataset because it contained the variables most relevant to the model in this study. The response rate for the 2014 survey was 55%. The survey is based on area probability sampling, which accounts for population size.

### 4.1. Dependent Variables

The study relied on two dependent variables: green pricing and green taxation. Each variable was based on a single item. For green pricing, respondents were asked, “How willing would you be to pay much higher prices to protect the environment?”; for green taxation, respondents were asked, “How willing would you be to pay much higher taxes to protect the environment?” [31]. The variables ranged from 1 to 5, with 1 indicating a lower level of support for green taxation or green pricing and 5 indicating a greater level of support. The average among respondents was 3.59 for green pricing and 3.33 for green taxation. 

### 4.2. Explanatory Variables

#### 4.2.1. Civic Morality

This measure is a multi-item measure based on six items, with each item ranging from 1 to 7. Respondents assessed how important the following six items were for them to be good citizens: voting regularly, tax compliance, watching government actions, acceptance of people with different views, purchasing ethical products, and helping worse-off Koreans. The measure was highly reliable (Cronbach’s alpha = 0.768). Scholars commonly rely on Cronbach’s alpha for the internal consistency of multi-item variables, and 0.7 is a threshold beyond which a variable is considered acceptable [32]. Further, the average was 5.57 above the mean of 5, indicating that respondents in general took civic morality seriously. As hypothesized earlier, individuals with a greater level of civic morality are expected to show a greater level of support for green pricing and taxation (H1 and H2).

#### 4.2.2. Political Participation

This measure is also a multi-item measure consisting of four items, with each item ranging from 1 to 4. Respondents assessed whether they participated in demonstrations, political rallies, forums on the Internet, and boycotting products. The average was 1.67 below the mean of 2.5, indicating that the respondents were generally passive in their level of political participation. The measure was also highly reliable (Cronbach’s alpha = 0.951). As hypothesized earlier, political participation serves as the moderator of the relationship between civic morality and green pricing and between civic morality and green taxation and was expected to strengthen the link between individuals’ sense of civic morality and their support for green pricing as well as green taxation (H3 and H4).

### 4.3. Controls

We also controlled for variables that may affect individuals’ support for green pricing and green taxation. First, perceived environmental threats were expected to be closely and positively associated with individuals’ support for the dependent variables. Individuals with a greater level of perception of environmental threats are more likely to consider environmental threats substantial than those with a weaker level of perception of such threats, motivating them to take protective actions, such as exhibiting environmentally friendly attitudes and supporting government actions that could alleviate their environmental concerns [33]. Several studies have demonstrated positive associations between perceived environmental threats and pro-environmental behavior [34,35]. For the measure, respondents were asked about their threat perceptions of air pollution produced by vehicles, air pollution caused by industry, pollution due to farming, water pollution, global warming, and genetically modified crops. The measure was highly reliable (Cronbach’s alpha = 0.984). 

Second, we expected political trust to be positively associated with individuals’ support for green pricing and taxation. Political trust is an evaluative measure based upon individuals’ expectations of government performance [36,37]. If they were satisfied with the government’s past performance, they would be more likely to place a greater level of trust in the government and what it would do in the future [38]. In other words, political trust serves as a heuristic, a mental shortcut by which individuals quickly lend their support to the government [39,40,41]. Thus, individuals with a greater level of political trust are likely to support taxation and higher prices for environmental protection. Studies have shown positive relationships between political trust and pro-environmental attitudes [42,43,44,45]. The measure is based on individuals’ levels of trust in the following four items: the central agencies, the President’s Office, the Korean Parliament, and the local government. The mean of the measure was 1.45, indicating that respondents were generally more distrusting of their government than trusting. 

Third, the model controlled for perceived local pollution. Individuals who perceive that their community is exposed to environmental threats are more likely to be motivated to support actions such as green pricing and taxation, which could ultimately strengthen environmental protection [46]. This measure consists of three items that assess respondents’ perceptions of air, water, and noise pollution in their community. The measure was highly reliable (Cronbach’s alpha = 0.989). Lastly, demographic variables, such as age, gender, income, and education levels, were accounted for in our model. Studies have shown that women in general are more likely to be environmentally friendly than men [47], and highly educated individuals are more likely to be aware of contemporary environmental issues, such as climate change, and be supportive of causes that may alleviate them [48]. Similarly, individuals with a higher level of income may prefer a healthier environment, which may drive them to support pro-environmental causes [40]. There is conflicting evidence regarding whether age is positively or negatively associated with pro-environmental behaviors [49]. The respondents’ ages ranged from 18 to 83 years. In terms of education, no respondent indicated a lack of formal education, and seven indicated a doctoral degree. Gender was coded as a dummy variable, with 1 indicating female and 0 indicating male. For income, 0 means no monthly income, whereas 21 means a monthly income of “more than KRW 10,000,000.” Table 1 describes the descriptive statistics of the variables used in this study.

### 4.4. Measurement Validity

To ensure that the measures included in the model were valid, we conducted a series of methodological inspections. Given that our study relied on a single dataset, it may be susceptible to common method variance. Thus, Harman’s single-factor test was implemented to assess whether one factor dominated the respondents’ responses to other factors included in the model. We found that one major factor explained only 17.99% of the covariance in the model, which was substantially below the commonly agreed-upon threshold. Furthermore, the respondents were guaranteed that their responses would remain anonymous, ensuring that they were not forced to produce desirable answers. Lastly, we conducted a confirmatory factor analysis to determine whether our model fit the data well. The indices produced are as follows: RMSEA = 0.047, CFI = 0.939, and TLI = 0.930. These indices meet generally accepted thresholds [50], indicating that the five-factor model fits the data well. 

## 5. Results

We relied on an ordered probit model for empirical analysis because the dependent variable was categorized as ordered values. The analysis also accounted for a weight that considered population representation as well as robust standard errors. The results are shown in Table 2. The analysis proceeded in two steps for each model. Model 1 considers when the dependent variable is green pricing, whereas Model 2 deals with green taxation as the dependent variable. For each model, the first step considers the direct relationship between the explanatory variables and the dependent variable; the second step focuses on the joint effects between the main explanatory variables and the dependent variable.

The results confirmed the four hypotheses. In terms of direct relationships (Step 1 of Model 1 and Model 2), individuals’ perceptions of civic morality were positively associated with green pricing as well as green taxation. Individuals with a greater degree of civic morality are likely to possess a measure of respect for fellow citizens [21,22]. They likely feel obligated to help fellow citizens and uphold the public interest that holds such citizens together [21,22]. Thus, these individuals may possess a high degree of acceptance of environmental attitudes that may alleviate the adverse effects of climate change. As a result, individuals with a greater degree of civic morality are highly likely to support green taxation and green pricing. 

More importantly, in terms of joint effects (Step 2 of Model 1 and Model 2), the study focused on whether political participation moderated the relationship between individuals’ sense of civic morality and their support for green pricing, as well as the relationship between individuals’ sense of civic morality and their support for green taxation. The results confirmed the hypotheses that political participation functions as the moderator and helps strengthen the positive link between civic morality and green pricing and between civic morality and green taxation. 

Scholars have noted that political participation facilitates self-actualization and self-competence with which individuals actively engage in reshaping political outcomes [7,9,17]. What makes such self-actualization possible is the mechanism of political self-efficacy [19,20]. A high degree of self-efficacy enables citizens to possess a high degree of self-competence, which results in active participation in resolving major environmental issues facing the world today. Imbued with a high degree of self-efficacy, individuals with a high degree of political participation are likely to tackle major environmental concerns. As mentioned earlier, because individuals with a greater degree of civic morality are already inclined to support environmental causes that uphold the public interest, and having a high degree of political participation produces functionally similar effects, political participation works as a moderator that helps strengthen individuals who are already inclined toward green taxation and green pricing. These theoretical reasonings explain why political participation in the model positively reinforces the link between civic morality and green taxation, as well as that between civic morality and green pricing.

In terms of the control variables, five variables were positively associated with both green pricing and green taxation. First, individuals with a greater level of perception of environmental threats were likely to support green pricing and green taxation that would facilitate protective actions toward remedying environmental degradation. Second, as expected, political trust worked as a mental shortcut that enabled individuals to support the government, which would rely on taxation and higher prices to protect the environment. Third, perceived local pollution was also positively associated with individuals’ support for green pricing and green taxation. Individuals who feel that their neighborhoods are polluted may want to see actions taken to ameliorate their environmental concerns by supporting the taxation and higher prices needed for such actions. In terms of demographic variables, income and education were positively associated with green pricing and green taxation. Individuals earning a higher income and possessing a greater level of education were more likely to recognize the grave environmental issues of the day and appeared to appreciate the need for environmental protection by supporting what was necessary to enhance it. 

**Figure 2:**
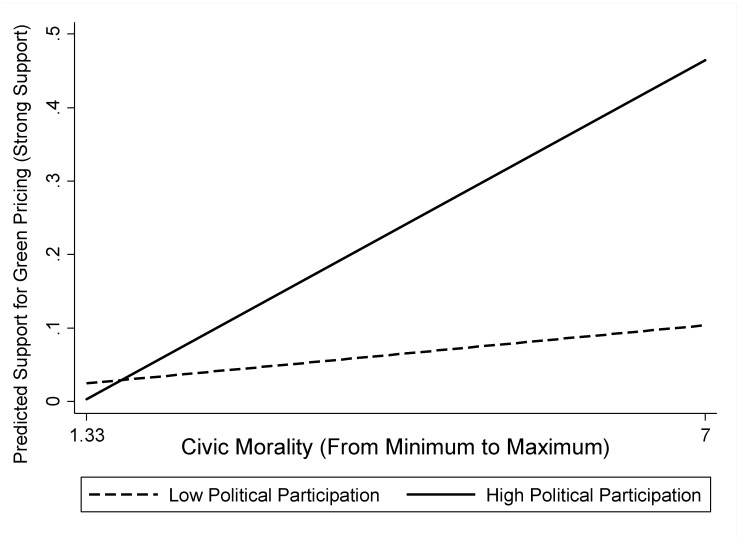
Predicted probabilities of strong support for green pricing.

**Figure 3:**
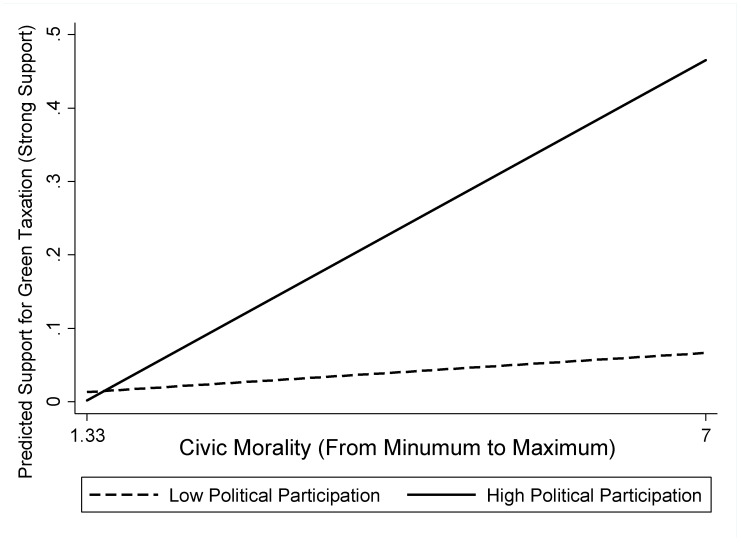
Predicted probabilities of strong support for green taxation.

Figure 2 and Figure 3 illustrate the joint effects of political participation and civic morality on individuals’ support for green pricing and taxation. The solid lines refer to a high degree of political participation (level of political participation = 4), and the dashed lines refer to a low degree of political participation (level of political participation = 1). As shown in Figure 2, when the degree of political participation was high, civic morality’s relationship with green pricing was not only positive in its direction but are also steep in its intensity. Compared with a lower degree of political participation, support for green pricing increased by more than 40% when individuals possessed a high degree of political participation. Similarly, as shown in Figure 3, when the degree of political participation was high, the slope denoting the link between civic morality and green taxation was positive and steep. Once again, individuals were likely to support green taxation by more than 40% when they possessed a high degree of political participation compared to a low degree of political participation. These two figures demonstrate the significance of the joint effects of political participation and civic morality and why it is important to study political participation as the moderator of the relationship between civic morality and green pricing, as well as between civic morality and green taxation.

## 6. Discussion 

In this study, we explored the factors that may influence green taxation and green pricing. Although these can be categorized as environmentally friendly behavior that may not directly resolve global climate challenges facing the world today, they can serve as steps that help mitigate the adverse consequences of human actions on climate. For instance, a review study showed that different cultural groups respond to climate challenges differently, and variations in values affect choices employed toward adaptations to climate change [51]. Adapting to and mitigating climate change also require significant collective action in a world of individuals and groups with complex, fragmented worldviews [52]. Although this study does not clarify how values or cultures are marshalled into a unified front in the fight against climate change, it touches upon a glimpse of what is possible against climate change when civic-minded individuals work together politically and actively participate in supporting environmental attitudes and behaviors (green taxation and pricing) that adapt to and mitigate climate change.

By exploring the moderation effect of political participation on the linkage between civic morality and environmentally friendly behavior, such as green taxation and green pricing, this study also helps address the value-action gap in prior research [10,11]. Values do not necessarily lead to actions; actions require a factor that links values to actions. This study identified political participation as a factor through which values (civic morality) can be amplified in connection with environmentally friendly behavior.

We explored factors that motivate people to support environmental taxation and higher pricing, focusing on the direct relationship between individuals’ perceptions of civic morality and their willingness to embrace green taxation and green pricing. The results demonstrated that a strong, positive link exists between civic morality and green taxation, as well as between civic morality and green pricing. The results showed that individuals’ sense of citizenship norms matters with respect to environmental causes that can help mitigate the environmental crisis facing the world today. Individuals with a high degree of citizenship norms are more likely to care about the public interest [5,6] and make necessary monetary sacrifices, such as higher taxation and pricing, if such sacrifices enhance the common good, such as environmental protection. 

More importantly, the study probed whether the positive relationship between individuals’ civic morality and their support for green taxation, as well as that between individuals’ civic morality and their support for green pricing, was moderated by their perceptions of participation in politics. As hypothesized, the results showed that individuals’ perceptions of political participation help strengthen the positive directions that individuals with a high level of civic morality may take toward their support for green taxation and green pricing. Individuals with a high degree of political participation may have a great deal of trust in their own self-control and self-competence to bring about political or social change that concerns communal causes [7,9,17]. Thus, they are likely to work assiduously toward communal causes, such as environmental protection. Given that highly civic-minded individuals are already likely to support green taxation and green pricing, active political participation would further reinforce the positive relationship between civic morality and green pricing, as well as between civic morality and green pricing, as civic morality and political participation serve as functional equivalents.

Overall, the results offer significant implications for policymakers concerned with environmental protection. First, individuals’ civic morality is a crucial ingredient for environmental causes. Although a well-devised policy is necessary for its effect, considering how citizens react to it should also be in the minds of policymakers. Scholars have noted that cultivating civic morality among citizens starts with schools [46,53], which are not limited to elementary schools but also extended to middle schools [54], high schools [55], and colleges [56]. 

From an early age, children are exposed to important worldviews and principles that bind society together. Children isolated from what constitutes being a citizen tend to cultivate skeptical views toward society and its policies [42,57]. The citizenship norms of children can be properly nurtured by providing them with opportunities to have unconstrained talks with others, work together with civic or politically oriented groups for a taste of political engagement, and participate in school-devised mock meetings and elections, where students learn how to vote and how to speak their minds in public settings [53]. As students become exposed to a variety of norms and rules by which to abide, they begin to cultivate a civic duty to hold society together. 

Second, political participation also works positively for environmental causes. Political participation helps strengthen individuals’ sense of self-control and self-competence, with which they help produce positive outcomes for communal outcomes, such as environmental protection [23,24]. Cultivating a sense of political participation works similarly to civic morality. In fact, exposing children to opportunities to discuss their thoughts and practice them in a variety of mock settings can also nurture their willingness to participate in politics [46,53]. Encouraging citizens to participate actively in political and administrative agendas may not be to policymakers’ liking, but doing so may eventually help them push for policies that meet citizens’ preferences. Public agencies can facilitate their policymaking by educating citizens about their policies and sending them materials to enhance their understanding of those policies. Properly engaged and informed citizens would be much more inclined to support causes related to environmental protection, even though such support entails sacrifices in their material interests in the form of higher taxation and pricing.

## 7. Conclusions

Severe weather and drastic climate change are increasingly upending people’s lives around the world. Recently, the state of Texas was virtually shut down for four days, and people who had never imagined such a winter storm and had never prepared for it infrastructurally suffered badly, with dozens of people finding themselves frozen to death [3,58]. These are some of the episodes surrounding the environmental crisis we are living in today. Although support for green taxation and green pricing may not constitute direct solutions to climate change, our study is one of the many small cultural, social, and political arsenals at our disposal to adapt to and mitigate climate change.

The limitations of this study must be acknowledged. First, the dependent variables consisted of single items. Although a high correlation exists between single-item variables and multi-item variables [59], the former are still less desirable due to their psychometric properties. Second, this study employed a single-year, cross-sectional dataset, with the data collected in a simultaneous timeframe. Thus, firm causality between the explanatory variables and the dependent variables may not be possible in this study. Third, political participation is an indirect measure to tap into political efficacy. Although political efficacy is a preferred measure to gauge individuals’ self-efficacy in political participation, it was not available in our dataset. Thus, the findings in this study need further verification by future studies that rely on actual political efficacy measures. Fourth, the dependent variables denote general attitudinal measures. Supporting green pricing and taxation does not necessarily mean that individuals will pay higher prices or taxes. Furthermore, these general measures do not indicate individuals choosing specific environmentally friendly actions, such as buying electric vehicles and recyclable products, boycotting environmentally harmful products, or banning unethical and unhealthy products regarding the environment and human health. Thus, future studies are needed to investigate whether the main explanatory variables used in this study are significantly associated with more specific, real-life, environmentally friendly outcomes. Lastly, our model did not account for the psychological states of the respondents in our model. Future studies should consider such aspects in understanding the link between civic morality and pro-environmental behavior, such as green taxation and green pricing.

## Figures and Tables

**Table 1:** Descriptive statistics.

Variables	N	Mean	S.D.	Min.	Max.
Green pricing	761	3.59	1.01	1	5
Green taxation	762	3.33	1.10	1	5
Civic morality	762	5.57	1.01	1.33	7
Political participation	762	1.67	0.66	1	4
Perceived environmental threats	762	3.62	0.63	1.67	5
Political trust	762	1.45	0.46	1	3
Perceived local pollution	762	2.45	0.63	1	4
Age	762	44.98	13.18	18	83
Female	762	0.41	0.49	0	1
Income	762	6.46	4.24	0	21
Education	762	4.00	1.39	0	7

**Table 2:** Regression results.

	Model 1: Support for Green Pricing				Model 2: Support for Green Taxation			
	Step 1		Step 2		Step 1		Step 2	
	Coef.	(S.E.)	Coef.	(S.E.)	Coef.	(S.E.)	Coef.	(S.E.)
Civic morality	0.19	0.05 ***	0.01	0.11	0.20	0.05 ***	0.00	0.10
Political participation	0.22	0.07 ***	−0.40	0.31	0.29	0.07 ***	−0.39	0.29
Civic morality × Political participation			0.11	0.07 **			0.12	0.05 **
Perceived environmental threats	0.22	0.09 **	0.23	0.09 ***	0.16	0.08 *	0.16	0.08 **
Political trust	0.23	0.09 **	0.24	0.09 ***	0.23	0.09 ***	0.25	0.09 ***
Perceived local pollution	0.26	0.08 ***	0.25	0.08 ***	0.14	0.07 *	0.14	0.07 *
Age	0.01	0.00 **	0.01	0.00 **	0.00	0.00	0.00	0.00
Female	−0.01	0.10	−0.02	0.10	−0.05	0.09	−0.06	0.09
Income	0.02	0.01 **	0.02	0.01 **	0.02	0.01 **	0.02	0.01 **
Education	0.08	0.04 **	0.09	0.04 **	0.10	0.03 ***	0.10	0.03 ***
τ_1_	2.19	0.49	1.22	0.69	1.95	0.45	0.89	0.64
τ_2_	2.97	0.49	2.01	0.69	2.84	0.45	1.78	0.65
τ_3_	3.48	0.50	2.52	0.70	3.32	0.46	2.27	0.65
τ_4_	5.32	0.52	4.36	0.71	5.02	0.47	3.98	0.66
Log likelihood	−901.37		−899.03		−990.86		−988.01	
Wald test	88.07		90.26		110.00		110.34	
Number of cases	761				762			

Note: * *p* < 0.1, ** *p* < 0.05, *** *p* < 0.01.

## Data Availability

The data used for this study are available at http://kgss.skku.edu/?page_id=39.
